# Supplementary figures and images for: The Role of Selenoproteins SELENOM and SELENOT in the Regulation of Apoptosis, ER Stress, and Calcium Homeostasis in the A-172 Human Glioblastoma Cell Line
Source: Biology (Basel). 2022 May 25;11(6):811. doi: 10.3390/biology11060811 (PMC9220170; doi:10.3390/biology11060811)

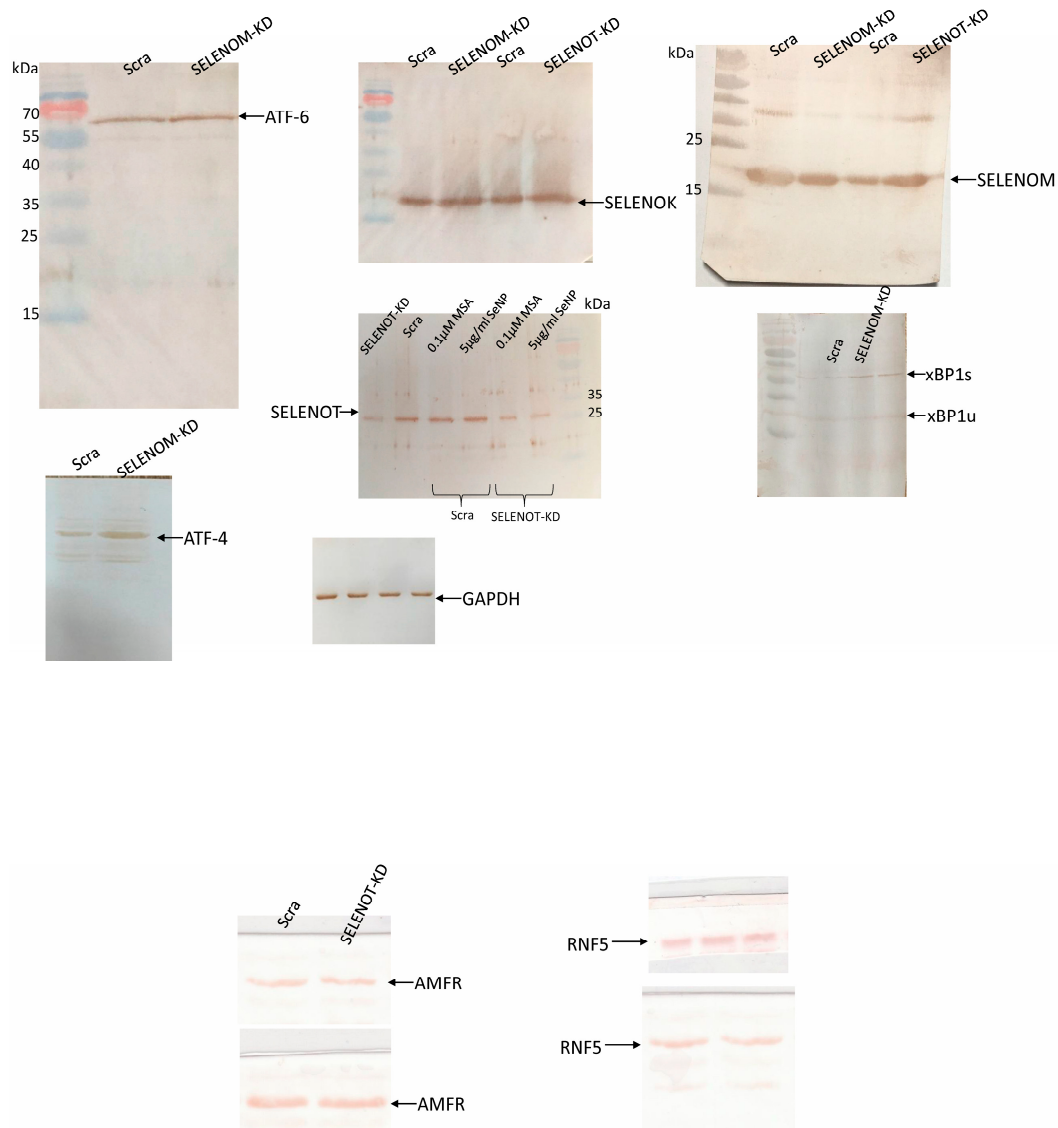

**Figure S1.** Original images of FullWestern Blot.

Supplement: Supplementary file 1 [file biology-11-00811-s001.zip › biology-1719286-supplementary.pdf]
